# A simple blind placement of the left-sided double-lumen tubes

**DOI:** 10.1097/MD.0000000000005376

**Published:** 2016-11-11

**Authors:** Zhi Jun Zong, Qi Ying Shen, Yao Lu, Yuan Hai Li

**Affiliations:** Department of Anesthesiology, First Affiliated Hospital of Anhui Medical University, Hefei, Anhui Province, P.R. China.

**Keywords:** double-lumen tube, one-lung ventilation, thoracic surgery

## Abstract

One-lung ventilation (OLV) has been commonly provided by using a double-lumen tube (DLT). Previous reports have indicated the high incidence of inappropriate DLT positioning in conventional maneuvers.

After obtaining approval from the medical ethics committee of First Affiliated Hospital of Anhui Medical University and written consent from patients, 88 adult patients belonging to American society of anesthesiologists (ASA) physical status grade I or II, and undergoing elective thoracic surgery requiring a left-side DLT for OLV were enrolled in this prospective, single-blind, randomized controlled study. Patients were randomly allocated to 1 of 2 groups: simple maneuver group or conventional maneuver group. The simple maneuver is a method that relies on partially inflating the bronchial balloon and recreating the effect of a carinal hook on the DLTs to give an idea of orientation and depth. After the induction of anesthesia the patients were intubated with a left-sided Robertshaw DLT using one of the 2 intubation techniques. After intubation of each DLT, an anesthesiologist used flexible bronchoscopy to evaluate the patient while the patient lay in a supine position. The number of optimal position and the time required to place DLT in correct position were recorded.

Time for the intubation of DLT took 100 ± 16.2 seconds (mean ± SD) in simple maneuver group and 95.1 ± 20.8 seconds in conventional maneuver group. The difference was not statistically significant (*P* = 0.221). Time for fiberoptic bronchoscope (FOB) took 22 ± 4.8 seconds in simple maneuver group and was statistically faster than that in conventional maneuver group (43.6 ± 23.7 seconds, *P* < 0.001). Nearly 98% of the 44 intubations in simple maneuver group were considered as in optimal position while only 52% of the 44 intubations in conventional maneuver group were in optimal position, and the difference was statistically significant (*P* < 0.001).

This simple maneuver is more rapid and more accurate to position left-sided DLTs, it may be substituted for FOB during positioning of a left-sided DLT in condition that FOB is unavailable or inapplicable.

## 1 Introduction

One-lung ventilation (OLV) is vital for open thoracotomy or video-assisted thoracoscopic surgery, because it facilitates lung exposure for the surgical procedure by collapsing the lung. OLV can be provided by using a double-lumen tube (DLT), an endobronchial tube, a bronchial blocker, or an endotracheal tube with a movable bronchial blocker. Among these methods, DLTs are the most commonly used among these methods. Previous reports have reported the high incidence of inappropriate DLT positioning in conventional maneuver,^[1,2]^ and inappropriate DLT positioning can produce adverse events during OLV. Therefore, proper positioning of DLTs is vital to performing OLV successfully. Generally, the use of flexible fiberoptic bronchoscope (FOB) is generally recommended for positioning of DLTs.^[3–5]^ However, the FOB is relatively expensive and may not always be available in clinical practice.^[6]^ Blood or mucus in the airway may also make it difficult to confirm the position of DLTs. Hence, to address the above shortcomings, we attempt to devise a *new and* simple method to perform blind left-sided DLTs intubation, which could be used when FOB is unavailable or inapplicable or when its use is difficult. The simple maneuver is a method that relies on partially inflating the bronchial balloon and recreating the effect of a carinal hook on the DLTs to give an idea of orientation and depth.

The aim of this study was to determine whether this simple maneuver can be used as a substitute for FOB verification or guidance in most conditions requiring left-sided DLTs.

## 2 Patients and methods

### 2.1 Study population

After obtaining approval from the medical ethics committee of First Affiliated Hospital of Anhui Medical University (No. 20130406, Prof. Runling Wang) and written consent from patients, 88 adult patients, belonging to ASA physical status grade I or II, aged 52 to 82 years, and undergoing elective thoracic surgery requiring a left-side DLT for OLV were enrolled in this study. Among the patients enrolled in the study, 66 were men and 26 were women. In terms of procedure, 22 underwent lobectomy, 58 underwent video-assisted thoracoscopic surgery, and 8 underwent exploratory thoracotomy. The patients were randomly allocated to the new maneuver group or the conventional maneuver group. Randomization (1:1) based on computer-generated codes. Patients excluded were those requiring right-sided DLT, presenting with an intraluminal lesion on the left bronchus or a very distorted anatomy of the tracheobronchial tree on chest radiograph, and the above conditions combined with upper respiratory tract infection, severe cardiopulmonary disease.

### 2.2 DLT intubation

Patients were monitored with electrocardiography, invasive arterial blood pressure, and pulse oxymetry. After induction of anesthesia with intravenous midazolam 0.1 mg/kg, propofol 1 to 2 mg/kg, sufentanil 0.5 μg/kg, and vecuronium 0.15 mg/kg, the patients were intubated with a left-sided Robertshaw DLT by the same thoracic anesthesiologist using one of the 2 intubation techniques. The size of the DLT was chosen according to the chest radiograph or chest computed tomography scan of the patient. Before use, the endobronchial cuff and endotracheal cuff were prepared with gas leakage measurement, well lubricated, and aspirated fully to collapse each pilot balloon totally.

**Figure 1:**
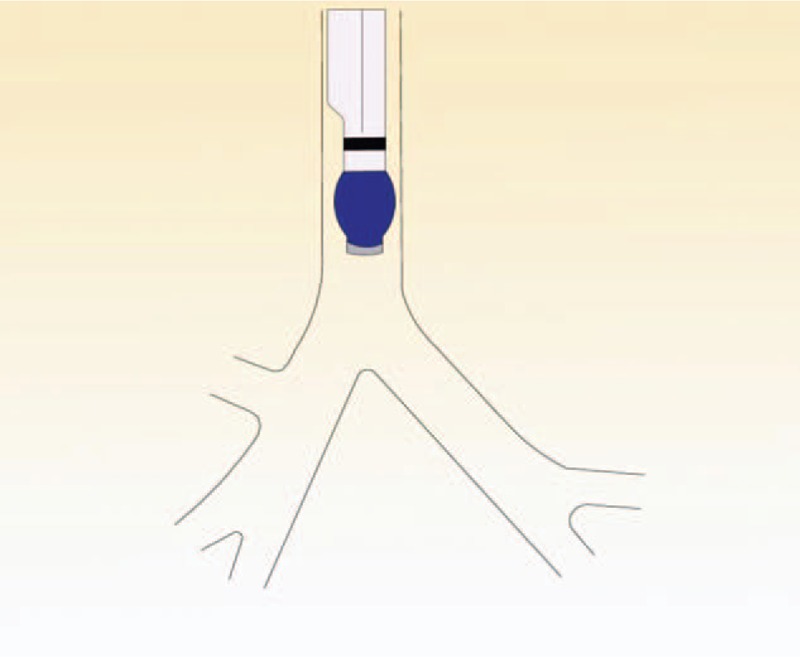
After the bronchial cuff passed the vocal cords, the stylet was removed but the laryngoscopy was not removed. The tube was rotated 90° toward the left, and the bronchial balloon was inflated with 5.0 to 7.0 mL of air. The tube was then advanced until resistance was felt.

**Figure 2:**
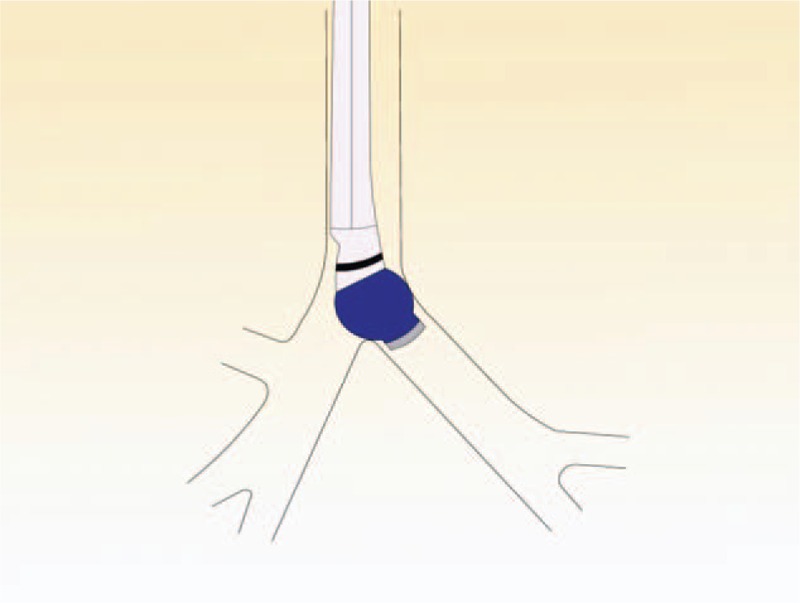
The lower edge of the inflated bronchial cuff was blocked just above the carina.

**Figure 3:**
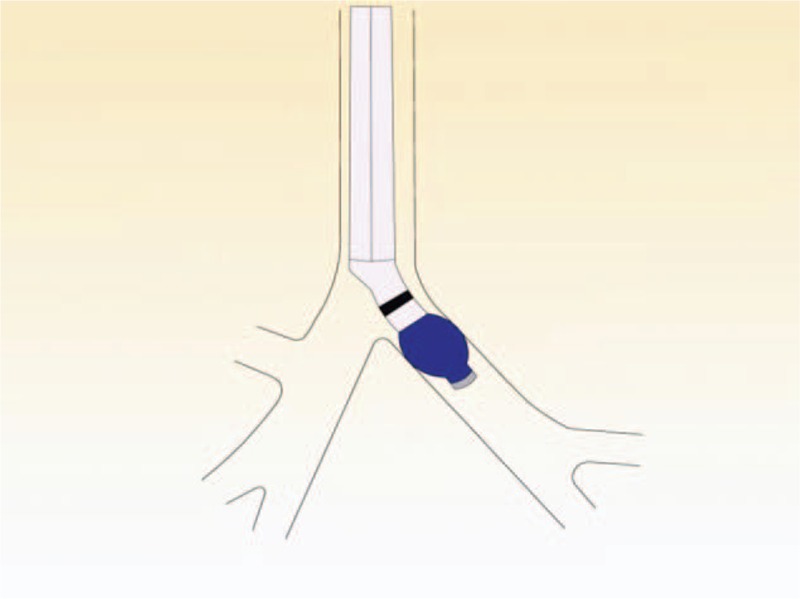
The inflated bronchial cuff was deflated; at this point, the upper edge of the bronchial cuff was expected to be just below the carina.

In the simple maneuver group, the tube was inserted by a thoracic anesthesiologist. The left-sided DLT (Broncho-cath, Mallinckrodt, Athlone, Ireland) was introduced into the glottis via direct laryngoscopy. The steps of the method are as follows. First, after the endobronchial cuff passed the vocal cords, the stylet was removed while the laryngoscopy was retained. Then, the endobronchial balloon was inflated with 5.0 to 7.0 mL of air after the tube was rotated 90° toward the left bronchus (Fig. 1). Second, the tube was advanced in steps while the breath sounds with each ventilation of the endobronchial lumen was auscultated, suddenly the breath sounds were present on the left chest and absent on the right chest. The change in breath sounds identified the position of the lower edge of endobronchial cuff as sitting just above the carina and at the entrance of the left main bronchus (Fig. 2). Third, the endobronchial cuff was deflated to advanced 15 mm forward, where the upper edge of endobronchial cuff was expected to be located just below the carina (Fig. 3). If breath sounds were present on the right chest after the endobronchial lumen was ventilated, the endobronchial lumen was considered as not having been inserted into the main left bronchus; hence, the tube was retracted into the trachea and the procedure repeated. If 2 attempts to advance the endobronchial lumen into the left bronchus were unsuccessful, FOB was used to guide the tube into the correct place.

In the conventional maneuver group, the tube was inserted blindly by the same thoracic anesthesiologist. The detailed steps are as follows. First, after the endobronchial cuff had passed the vocal cords, the stylet was removed, and the tube rotated 90° toward the left and advanced until a certain resistance is encountered; Second, after successful blind intubation, the anesthesiologist inflated the endobronchial cuff with the appropriate volume of air (2–3 mL), observed chest wall movements, auscultated both lungs before and after selective clamping of endobronchial or tracheal lumen, and checked lung compliance by manual ventilation to verify the correct position of the DLT.

After intubation of each DLT and with the patient still in the supine position, flexible bronchoscopy (ultra-slim 2.8-mm diameter Olympus video bronchoscope) was performed by another anesthesiologist, who was not aware of the intubation methods to assess DLT position.

### 2.3 Variables

Optimal position was defined as the endobronchial cuff located immediately below the carina with a clear view of the left subcarina and unobstructed left upper and lower bronchi. After bronchoscopic assessment, the DLT was either secured in its optimal position or repositioned according to the bronchoscopic assessment under direct visualization with a video bronchoscope. The assessment of the DLT position was recorded in the anesthesiologist's chart.

The time taken for initial tube placement with the patient in supine position was defined as the time the tube passed the vocal cords until a subjectively satisfactory placement of the endobronchial lumen was achieved.

Immediately before the start of surgery, patients were turned to the lateral position, and flexible bronchoscopy was performed to verify whether the DLT was still well placed. Data obtained from the second bronchoscopic evaluation were not used for the analysis because the aim of the study was to compare the rate of correct blind insertion of DLT in the simple maneuver with that in the conventional maneuver immediately after intubation.

The variables recorded in this study were the number of optimal position and the time required to place DLT in the correct position.

### 2.4 Statistical analysis

Sample size was based on a pilot study during which we measured the successful rate of DLT intubation with the new maneuver as well as a previous study. We determined that to detect a 30% absolute difference in the successful rate of DLT intubation with the simple maneuver and assuming a type I error protection of 0.05 and a power of 0.80 34 patients were needed in each group. However, because of the difficult setting and the potential risk of failure to intubate despite careful preparation, the sample size was enlarged and 88 patients were enrolled (44 per group) into the trial.

Variables with continuous distribution (such as age, height, weight, time for DLT placement, and time for FOB confirmation) were summarized using means and standard deviations. Group comparisons were based on the results of the independent *t* test. Variables with discrete distribution were presented using counts and percentages, and compared across groups using the Pearson Chi-square test or Fisher exact test. All statistical analyses were performed using the SPSS 17.0 software package and a *P*-value of less than 0.05 was considered statistically significant.

## 3 Results

Both groups were comparable with respect to age, weight, height, sex, ASA physical status grade, and DLT selected size (*P* > 0.05) (Table 1).

**Table 1:**
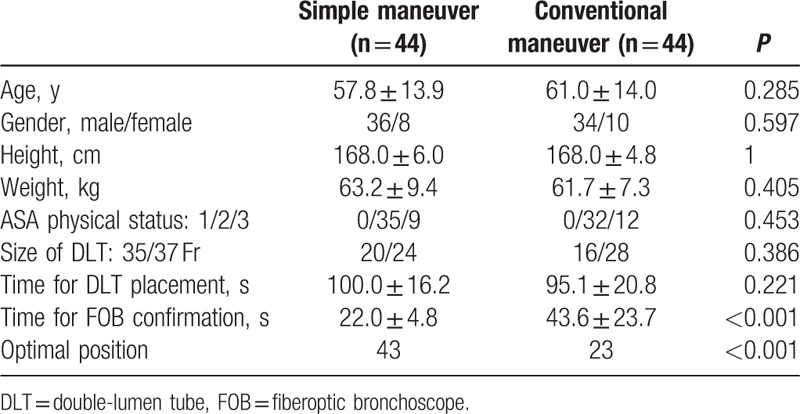
Patients characteristics and details of the double-lumen tube inserted.

The mean of DLT intubation time was 100 (16.2) seconds in the new maneuver group and 95.1 (20.8) seconds in the conventional maneuver group. The difference was not statistically significant (*P* > 0.05). The mean of FOB verification time was 22 (4.8) seconds in the new maneuver group and was statistically faster than that in the conventional maneuver group [43.6 (23.7) seconds, *P* < 0.05] (Table 1).

Nearly 98% of the 44 intubations in the new maneuver group was considered as being in an optimal position while only 52% of the 44 intubations in conventional maneuver group were in the optimal position. The difference was statistically significant (*P* < 0.05) (Table 1).

## 4 Discussion

The DLT is commonly used in thoracic procedures for OLV, because it can isolate and collapse the lungs selectively during the procedures. Accurate positioning of DLT is important because a misplaced DLT could jeopardize the operation or injure the patient.

Red rubber DLT is the oldest type of DLT used for OLV. This type of DLT is easy to position because it has a carinal hook. However, the intubation method is complex, causing injury to the patient's airway and raising the risk of cross infection when used repeatedly.^[7]^ Hence, a new kind of DLT with no carinal hook, the Robertshaw DLT, is largely applied in favor of a less traumatic technique.

However, without the carinal hook, the DLT is difficult to position correctly. Auscultation method is often used to position the DLT, but this method is more subjective and has a lower rate of successful intubation compared with Red rubber DLT. Hence, anesthesiologists have been attempting to improve the intubation methods. A bronchofibroscope is a good instrument to position the DLT because of its high incidence of success. However, a bronchofibroscope also has many disadvantages. First, a bronchofibroscope is expensive and thus is not widely used or not enough in anesthesia departments. Many anesthesia departments lack smaller bronchofibroscope for positioning 26 or 28 Fr DLT used in children.^[8]^ Second, blood or mucus in the airway and anatomical distortion can make visual confirmation of DLT position difficult or impossible. Third, a bronchofibroscope does not guarantee success because positioning problems and serious complications can still occur even when an FOB is used.^[9,10]^

In 1992, Russell^[11]^ introduced a simple way for blind placement of the DLTs. However, he did not make a statistical analysis on the difference between this simple way and the conventional method. In clinical practice the author also pondered on this simple way to position the left-sided DLT, in which the inflated endobronchial cuff was used as the “carinal hook,” and in preliminary experiment, the method obtained a high success rate. Therefore, we devised this clinical test expecting to position the left-sided DLT successfully without the aid of FOB.

Our study shows that the position of a left-sided DLT was in optimal in 43 of 44 in the simple maneuver group. Therefore, the simple maneuver is more effective than the conventional maneuver. In the simple maneuver group, the inflated endobronchial cuff of the DLT functioned as a carinal hook. Therefore, the DLT can be positioned more accurately. In the conventional maneuver group, the position of the left-sided DLT was in optimal in only 23 of 44, which is similar to previous studies. Smith et al^[1]^ reported that 48% of blindly positioned DLTs in 23 patients were malpositioned, whereas Alliaume et al^[2]^ reported that blindly placed left-sided DLTs required repositioning in 78% of 18 patients. These results could be because using auscultation may make it difficult at times to distinguish breath sounds and transmitting sounds from other parts of the lung in the conventional method. Only 1 case in each group was intubated into the right bronchus which was subsequently corrected by FOB.

Intubation time in the simple maneuver group was similar to that in the conventional maneuver group. However, the time needed to check the position of DLTs in the simple maneuver group was less than that in conventional maneuver group. This result is mainly because this simple method has higher optimal intubation rate and DLTs position after first intubation and scarcely required readjustment.

The width of the left bronchus is directly proportional to tracheal width. The left bronchus width is estimated by multiplying the tracheal width by 0.68.^[12]^ Therefore, if we want to make the inflated endobronchial cuff of DLT not only pass through trachea but also be blocked at the carina level, we can inflate the endobronchial cuff until the width of the cuff is about 0.8 times that of tracheal width (inflation can be accomplished before intubation and the amount of the air should be recorded). The same reason applies to why we inflate 5 to 7 mL air into the endobronchial cuff just after passes through vocal cords during the intubation.

The length of the left inflated bronchial cuff is 15 mm, thus, the endobronchial lumen with deflated endobronchial cuff continued to be advanced 15 mm from the point where the inflated endobronchial cuff was blocked, at which point the upper edge of the endobronchial cuff was expected to be just below the carina.

The left main bronchus is longer than the right bronchus, and a left DLT can be applied for both right- and left-sided procedures.^[13]^ Hence, we only studied left-sided DLT.

Although patient height can be used as basis for selecting a DLT,^[14]^ the correlation between airway size and height is extremely poor.^[15]^ Tracheal and endobronchial dimensions can be measured from the chest radiograph or chest computed tomography scan.^[16–19]^ In this study, all DLT sizes were selected according to the chest radiograph or chest computed tomography scan of the patient.

All DLTs should be lubricated well before intubation to ensure convenient operation and decrease bronchus injury during intubation.

The sample size of this study was relatively small and we did not apply this maneuver to right-sided DLT intubation. DLT displacement after patients turn to lateral position was also not investigated.

In conclusion, this simple maneuver is more rapid and more accurate to position left-sided DLTs than the conventional maneuver. Hence, the simple maneuver can be used as a substitute for FOB when positioning of a left-sided DLT if FOB is unavailable or inapplicable.
