# A simple blind placement of the left-sided double-lumen tubes: Erratum

**DOI:** 10.1097/MD.0000000000006196

**Published:** 2017-02-17

In the article “A simple blind placement of the left-sided double-lumen tubes”,^[1]^ which appeared in Volume 95, Issue 45 of *Medicine*, the Methods section incorrectly stated the numbers of men and women who participated in the study. The statement should have read as follows:

Among the patients enrolled in the study, 70 were men and 18 were women.
